# Supplementary material for: Development and Optimization of a Selective Whole-Genome Amplification To Study Plasmodium ovale Spp
Source: Microbiol Spectr. 2022 Sep 13;10(5):e00726-22. doi: 10.1128/spectrum.00726-22 (PMC9602584; doi:10.1128/spectrum.00726-22)
Supplement: Supplemental file 1 — Fig. S1 to S8, Tables S1 to S3, and Table S6. Download spectrum.00726-22-s0001.pdf, PDF file, 1.7 MB [file spectrum.00726-22-s0001.pdf]

Supplemental Material

I. Supplemental figures

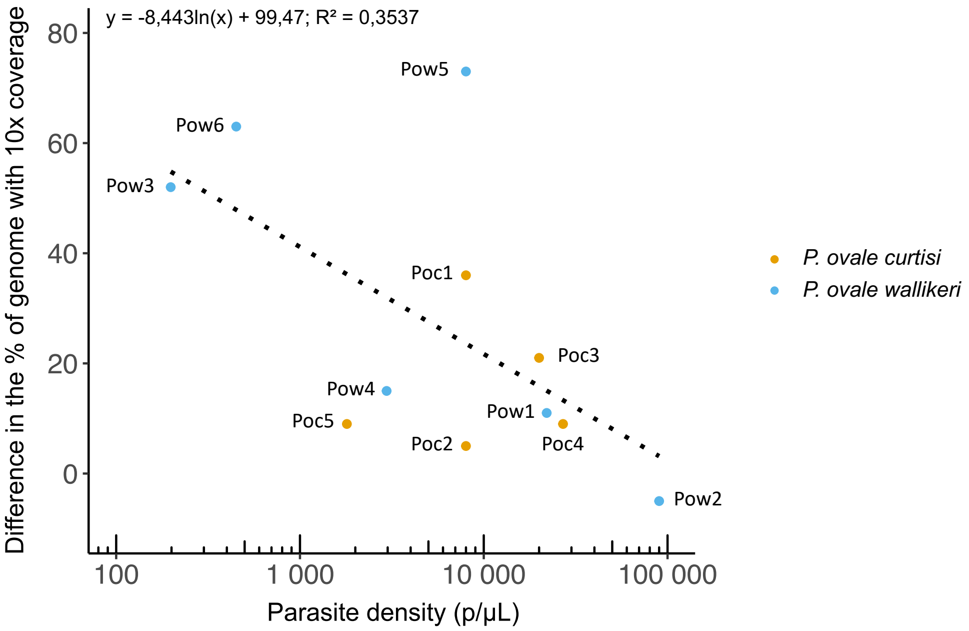

Figure S1. Difference between the sWGA+McrBC and the sWGA conditions for the percentage of the genome with a depth of coverage ≥ 10x for *P. ovale curtisi* (in light brown) and *P. ovale wallikeri* (in blue). Sample identification is indicated.

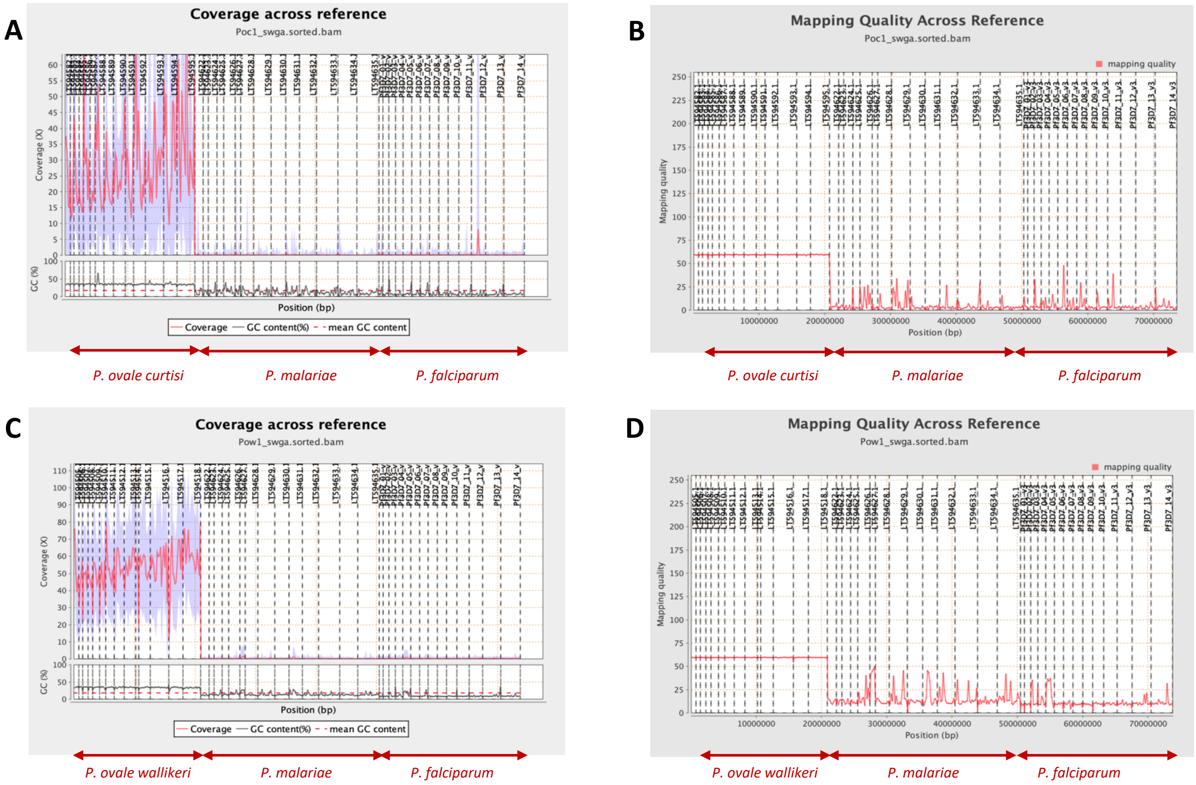

Figure S2. Coverage and mapping quality of *P. ovale curtisi* (Poc1, A and B) and *P. ovale wallikeri* (Pow1, C and D) short reads generated by sWGA against a concatenate genome of *P. malariae* (PmUG01, LT594622 to LT594635), *P. falciparum* (Pf3D7, Pf3D7_01_v3 to Pf3D7_14_v3) and *P. ovale curtisi*/*P. ovale wallikeri* (PocGH01 or PowCR01, LT594582 to LT594595 or LT594505 to LT594518). Plots were generated using Qualimap (v2.2.1).

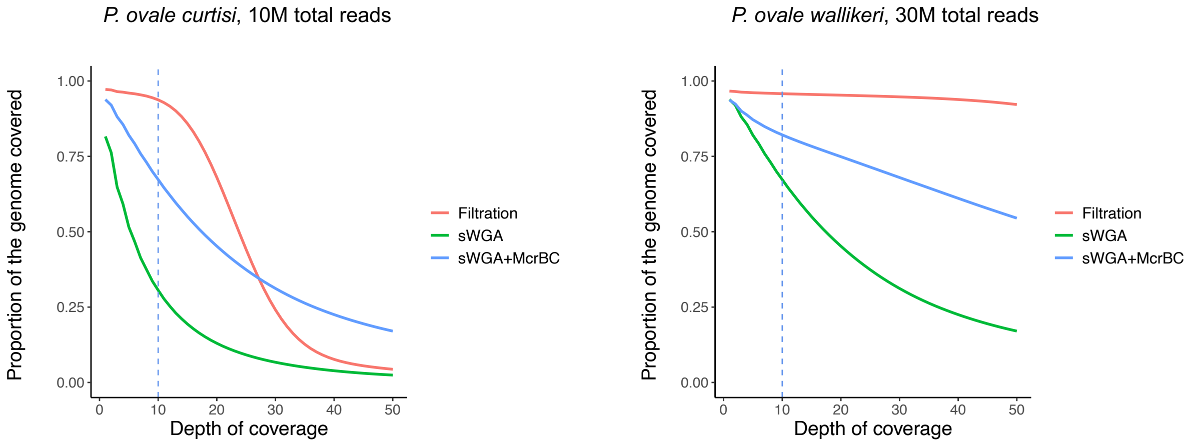

Figure S3. Proportion of the genome covered with or without the McrBC endonuclease enzyme and after filtration with MN2100ff cellulose for both A) *P. ovale curtisi* and B) *P. ovale wallikeri*. The blue dashed line represents a depth of coverage of ten.

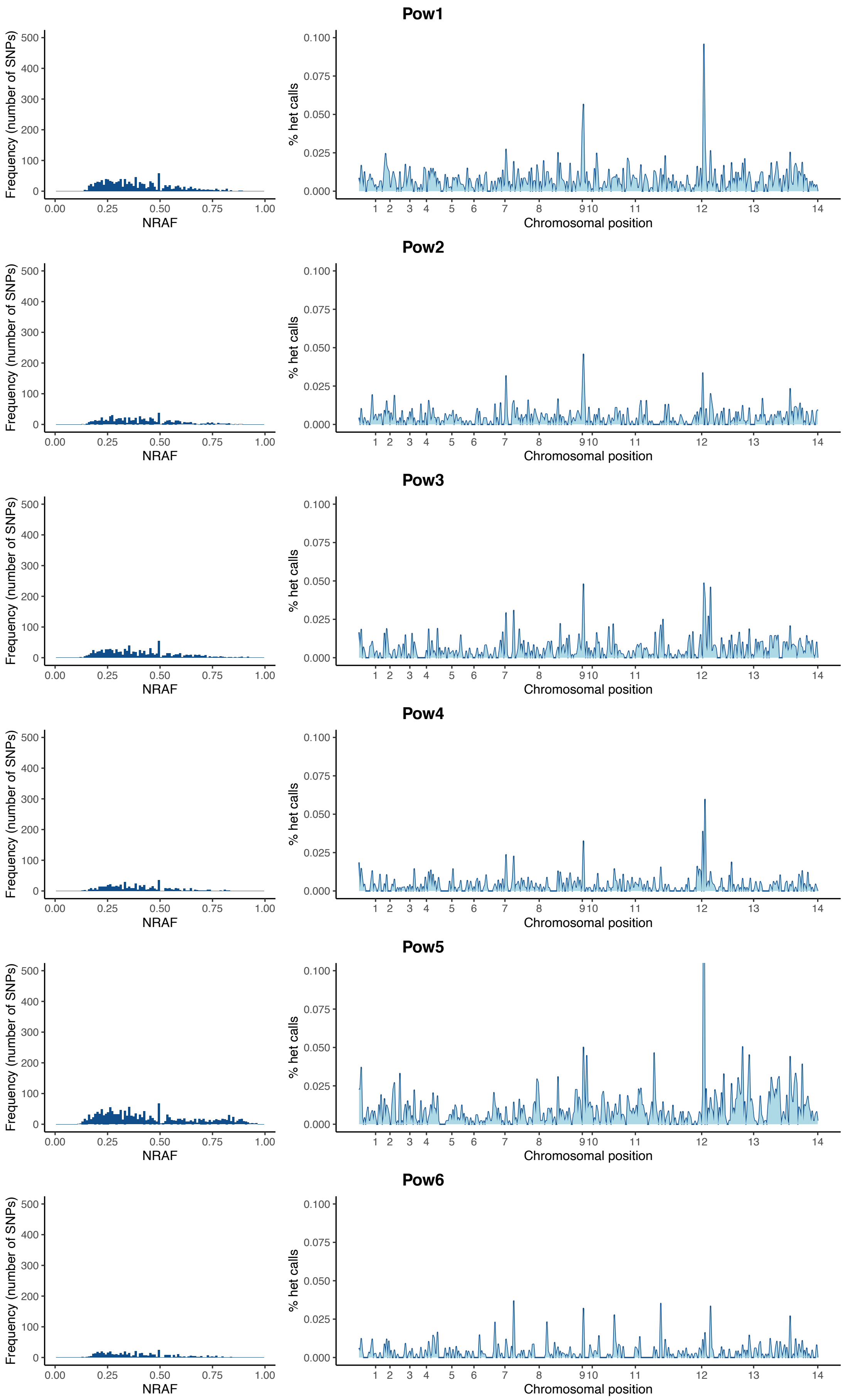
Pow1
Pow2
Pow3
Pow4
Pow5
Pow6
Frequency (number of SNPs)
0
100
200
300
400
500
0.00
0.25
0.50
0.75
1.00
NRAF
% het calls
0.000
0.025
0.050
0.075
0.100
1
2
3
4
5
6
7
8
9 10
11
12
13
14
Chromosomal position

Figure S4A. NRAF and percentage of heterozygote calls across the chromosomes for *P. ovale wallikeri* isolates sequenced with the sWGA + McrBC approach. NRAF stands for Non-Reference Allele Frequency and % het calls stands for percentage of heterozygote calls.

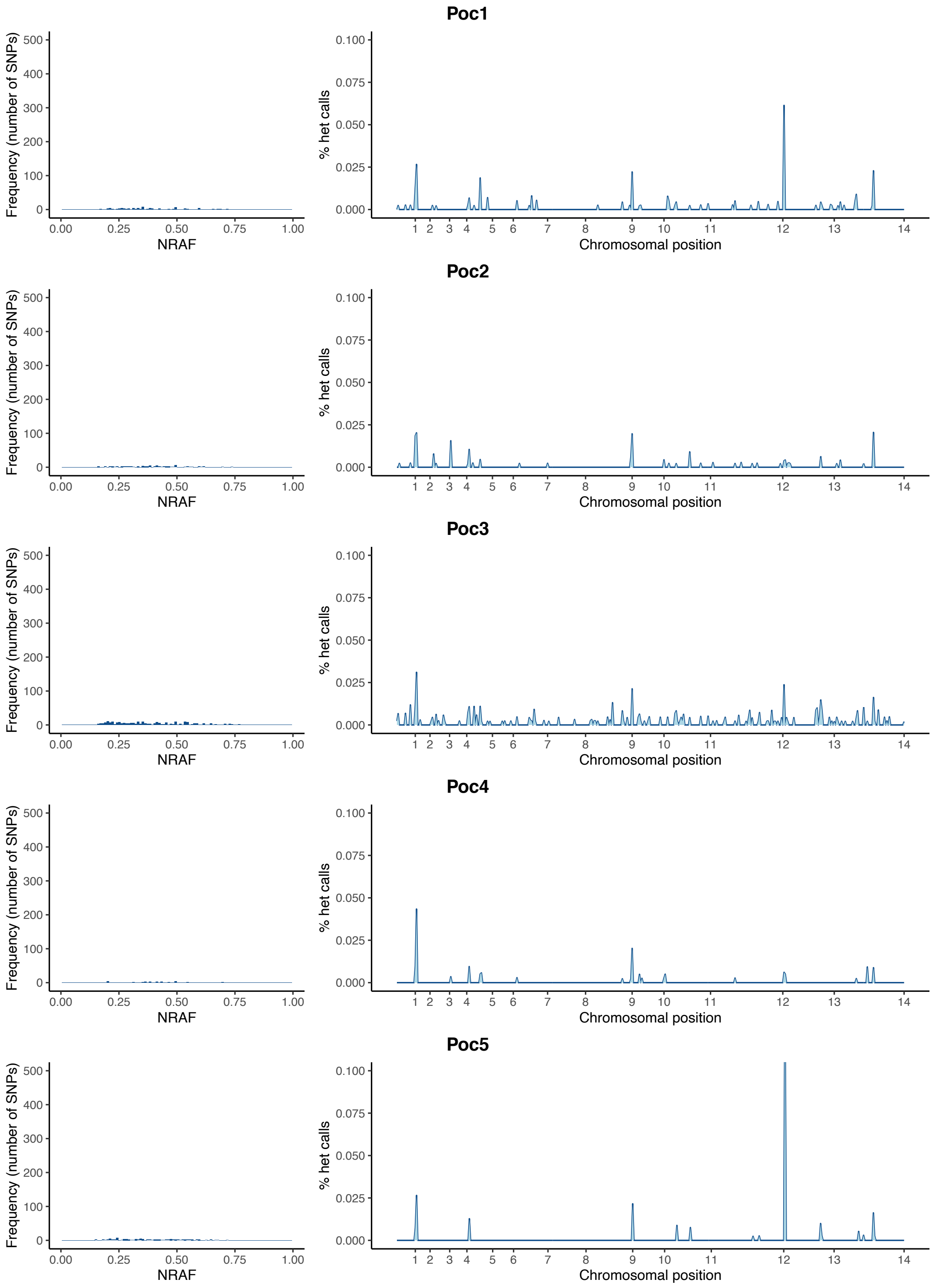

Figure S4B. NRAF and percentage of heterozygote calls across the chromosomes for *P. ovale curtisi* isolates sequenced with the sWGA + McrBC approach. NRAF stands for Non-Reference Allele Frequency and % het calls stands for percentage of heterozygote calls.

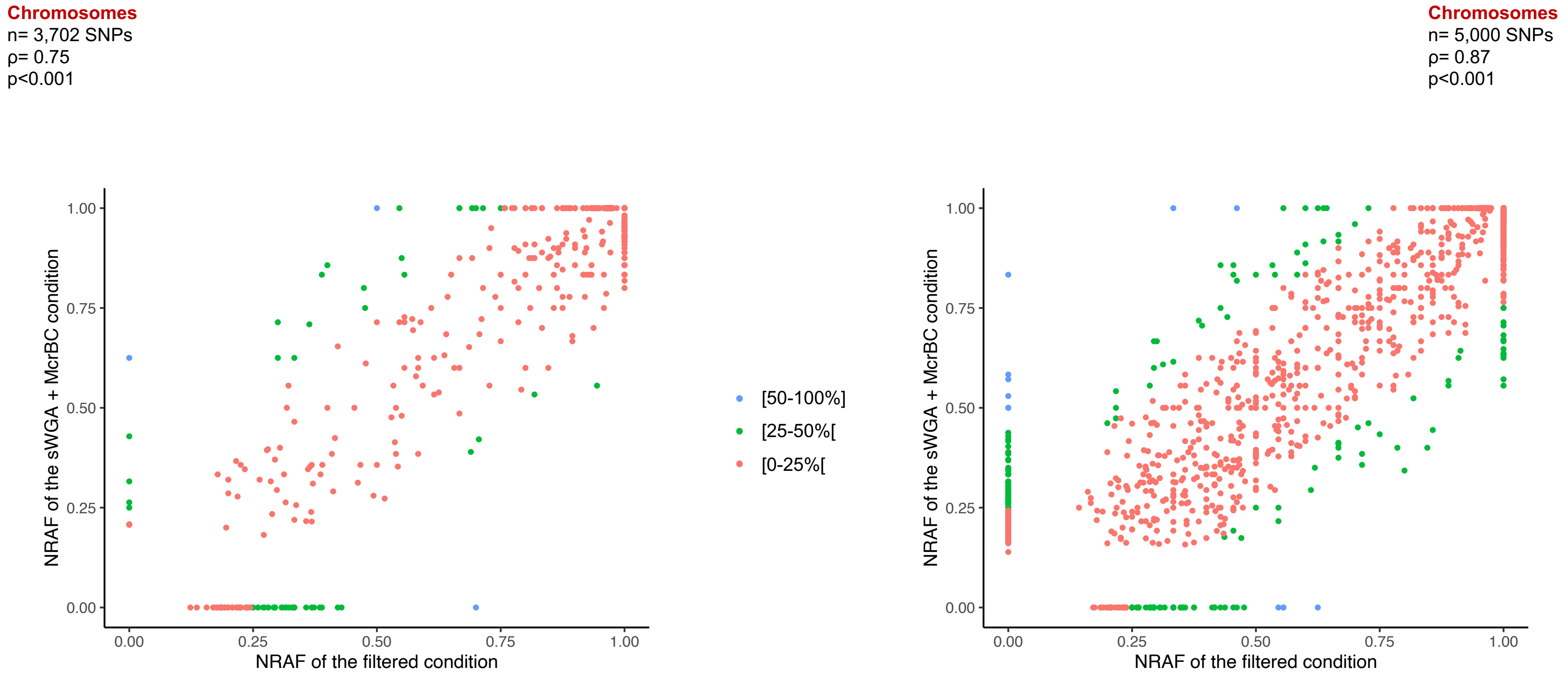

Figure S5. Non-Reference Allele Frequency (NRAF) correlation for the chromosomes' SNPs between the filtration and the sWGA + McrBc method for both *P. ovale curtisi* and *P. ovale wallikeri*. Red points indicate a difference of the NRAF between the two conditions < to 25%, green points a difference between 25 to 50% and blue points a difference between 50 to 100%. The number of SNPs tested (n), the Spearman coefficient (ρ) and p-value of the Spearman rank test correlation are indicated.

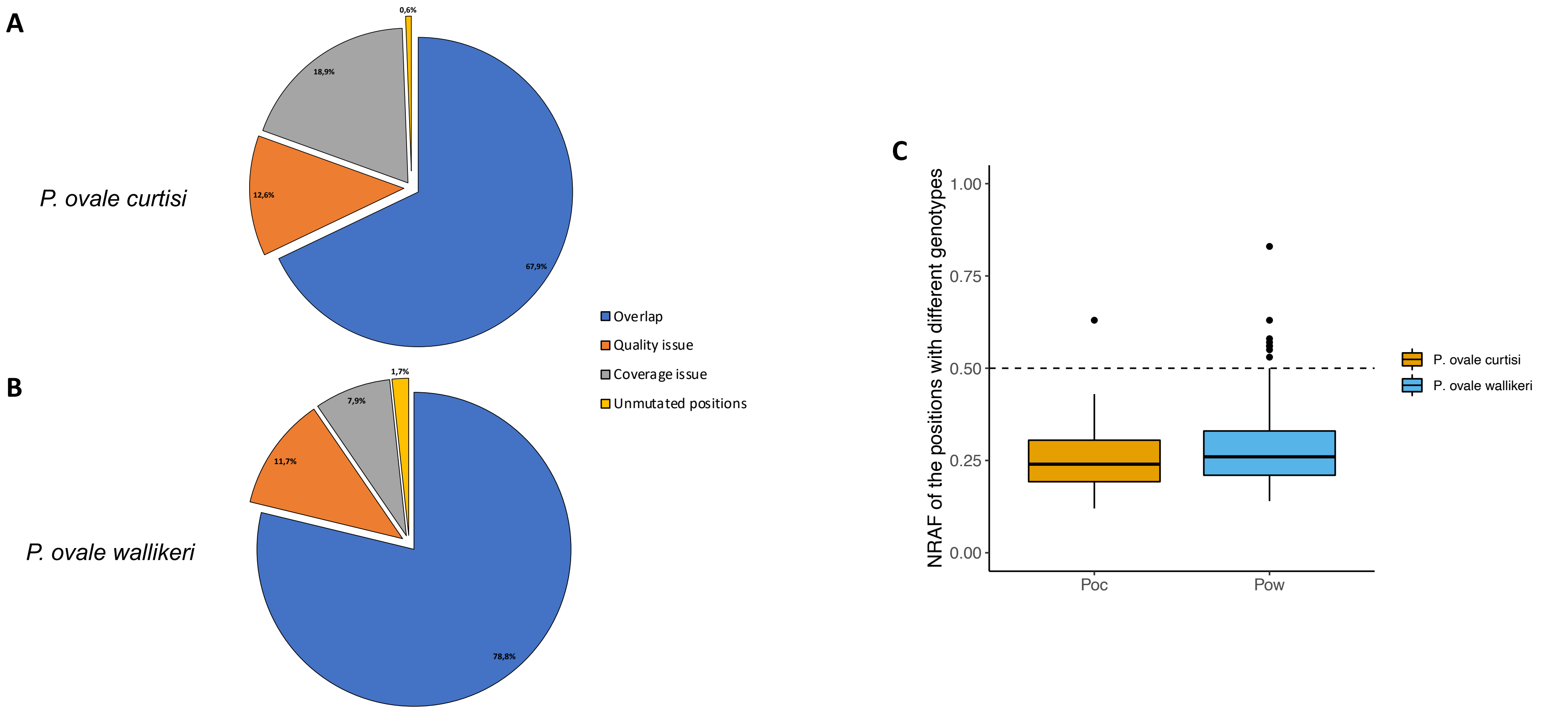

Figure S6. A and B. Comparison of the SNPs obtained with the sWGA and the filtration methods for both *P. ovale curtisi* (A) and *P. ovale wallikeri* (B). Overlap category correspond to identical SNPs obtained with both methods. Quality issue correspond to SNPs obtained for one method that did not pass the quality filters for the other method. Coverage issue correspond to SNPs obtained for one method that did not pass the depth filters for the other method. Unmutated positions correspond to positions with SNPs (heterozygote or homozygote mutant) for one method and no mutation with the other method. **C**. NRAF of the positions with different genotypes between sWGA and filtration (wild-type to heterozygote or homozygote mutant). Poc stands for P. ovale curtisi and Pow stands for P. ovale wallikeri.

```
CLUSTAL O(1.2.4) multiple sequence alignment

PocGH01_05028400       -MEELADVFDIYAICACCKVSKEGDWKKSESYSNSTFRGIGNKGILPWKYNSVDISYFSS 59
PowCR01_050023500      -MEEVADVFDIYAICACCKVSKEGDWKKSESFSSSTFRGIGNKGILPWKCNSVDISYFSS 59
Pf3D7_0417200          MMEQVCDVFDIYAICACCKVESKNEGKKNEVFNNYTFRGLGNKGVLPWKCNSLDMKYFCA 60
PvP01_0526600          -MEDLSDVFDIYAICACCKVAPTSEGTKNEPFSPRTFRGLGNKGTLPWKCNSVDMKYFRS 59
PmUG01_05034700        -MEDLADIFDIYAICACCKVPNQGEGKKNEIFSTKTFRGLGNKGCLPWKSNSLDMKYFRS 59
                        **::.*:************   .: .*.* :.  ****:**** **** **:*:.** :

PocGH01_05028400       VTTYVNEWNYNKLKYKREKYLEKDISNDKKKV----DVINIAHISKKLQNVVVMGRSSWE 115
PowCR01_050023500      VTTYVNEWNYKKLKYKREKYLEKDISNDKKKV----DVINIAPISKKLQNVVVMGRSSWE 115
Pf3D7_0417200          VTTYVNESKYEKLKYKRCKYLNKETVDN----------VNDMPNSKKLQNVVVMGRTSWE 110
PvP01_0526600          VTTYVDESKYEKLKWKRERYLRMEASQGGGDNTSGGDNTHGGDNADKLQNVVVMGRSNWE 119
PmUG01_05034700        VTTYVNEMKYKKLKYKREKYLEKEISNENSSTVF---ENISLLSSSKLQNVVVMGRSNWV 116
                       *****:* :*:***:** :**. :  :                 :.**********:.*

PocGH01_05028400       SIPKSYKPLANRINVVLSSTLKKEDVKEDIFIMKSMDEVLLLLKKLYYYKCFIIGGAGVY 175
PowCR01_050023500      SIPKSYKPLANRINVVLSSTLKKEDVKEDIFIMKSMDEVLLLLKKLYYYKCFIIGGAGVY 175
Pf3D7_0417200          SIPKKFKPLSNRINVILSRTLKKEDFDEDVYIINKVEDLIVLLGKLNYYKCFIIGGSVVY 170
PvP01_0526600          SIPKQYKPLPNRINVVLSKTLTKEDVKEKVFIIDSIDDLLLLLKKLKYYKCFIIGGAQVY 179
PmUG01_05034700        SIPKQYKPLPNRINVVLSRTLKKEDVKEDIFIINNMDQLVLLLKKLNYYKCFIIGGAIVY 176
                       ****.:*** *****:** **.***..*.::*:..::::::** ** *********: **

PocGH01_05028400       KECLERNLIKQVYLTRINNTYECDVFFPEMDKNTFQITSVSEVYSSNGTTLDFLIYSRKK 235
PowCR01_050023500      KECLERNLIKQIYLTRINNTYECDVFFPDMDENAFQITSVSEVYSSNGTTLDFLIYSRKK 235
Pf3D7_0417200          QEFLEKKLIKKIYFTRINSTYECDVFFPEINENEYQIISVSDVYTSNNTTLDFIIYKKTN 230
PvP01_0526600          RECLSRNLIKQIYFTRINGAYPCDVFFPEFDESQFRVTSVSEVYNSKGTTLDFLVYSKVG 239
PmUG01_05034700        KECLERNLIKQIYFTRINNVYECDVFFPEIDENVFQITSVSDVYTSNCTSLDFVIFSKRK 236
                       :* *.::***::*:****..* ******::::. ::: ***:**.*: *:***:::.:
```

Figure S7. Multiple alignment with Clust O (v.1.2.4) of dihydrofolate reductase domain of the *bifunctional dihydrofolate reductase-thymidilate synthase* genes of *P. ovale curtisi* (PocGH01_05028400), *P. ovale wallikeri* (PowCR01_050023500), *P. falciparum* (Pf3D7_0417200), *P. vivax* (PvP01_0526600) and *P. malariae* (PmUG01_05034700). Red arrows indicate mutations of interest in *P. ovale curtisi* (A15S), green arrows in *P. ovale wallikeri* (F57L and S113N) and blue arrows in both *P. ovale* spp. species (S58R).

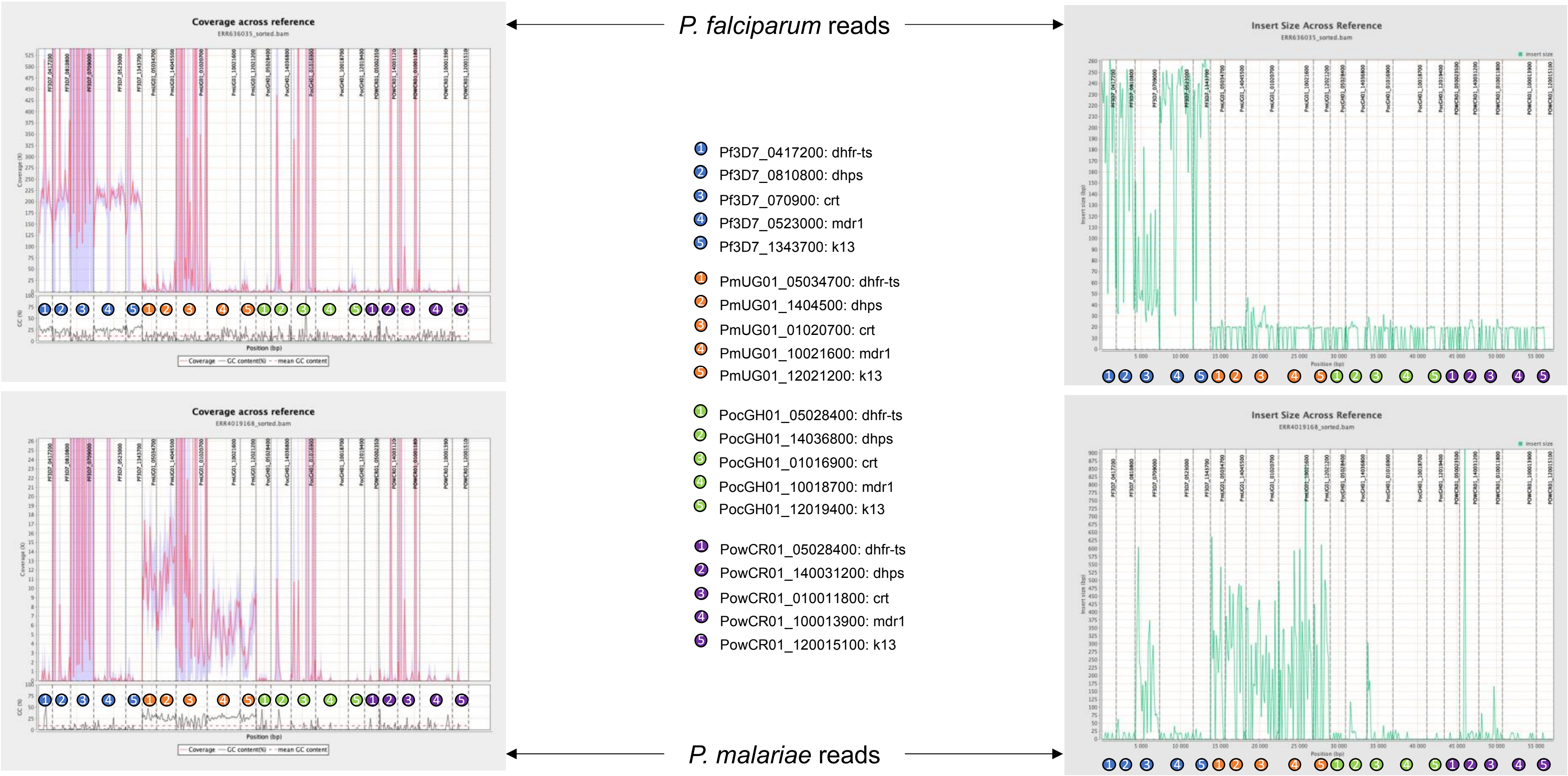

Figure S8. Coverage across reference and insert size across reference for ERR636035 (*P. falciparum*, at the top) and ERR4019168 (*P. malariae*, at the bottom) on a concatenate sequence composed of five known resistance genes of *P. falciparum* and the orthologous genes of *P. malariae*, *P. ovale curtisi* and *P. ovale wallikeri*. Plots were generated using Qualimap.

II. Supplemental tables

Isolat	Species	Before filtration			After filtration			% of mapped reads	Mean coverage	Percentage of genome with		
		Ct ovale	Ct humain	ΔCt	Ct ovale	Ct humain	ΔCt			1x cov	5x cov	10x cov
Poc1	*P. ovale curtisi*	25	21	4	28	30	-2	74	93	98	97	97
Pow1	*P. ovale wallikeri*	24	20	4	24	32	-8	95	99	97	96	96

Table S1. qPCR Ct before and after filtration and WGS data quality of the two isolates sequenced after filtration with the MN2100ff cellulose. cov stands for coverage.

	Genome size (s)	Poc primers' set		Pow primers' set	
		**Number of binding (n)**	**s/n (bp)**	**Number of binding (n)**	**s/n (bp)**
P. ovale spp.	33 Mbp	4,551	7,251	4,986	6,618
P. falciparum 3D7	23 Mbp	2,127	10,814	2,267	10,146
P. malariae UG01	34 Mbp	4,218	8,061	4,791	7,097
P. vivax P01	29 Mbp	2,760	10,507	2,790	10,394
Human GRCh38	2948 Mbp	23,005	128,151	25,492	115,644

Table S2. Number of primers' binding and Genome size/Number of binding ratio for the PocGH01 or PowCR01, Pf3D7, PmUG01, PvP01 and GRCh38 genomes for each Poc and Pow primers' sets.

Sample	Without methylation digest						
	Reads (m)	% of mapped reads	Mean coverage	Normalized coverage	Percentage of total genome with		
					1X coverage	5X coverage	10X coverage
Poc1	7	73	11,9	1,70	82	52	31
Poc2	34	44,9	36,2	1,06	98	93	80
Poc3	37	48,2	39	1,05	96	81	62
Poc4	32	55,2	45	1,41	95	77	58
Poc5	30	45,4	30,3	1,01	92	70	52
Pow1	34	41,7	37,3	1,10	92	81	71
Pow2	36	56,7	57,7	1,60	97	94	90
Pow3	32	25,4	10,1	0,32	88	61	36
Pow4	26	36,5	26,4	1,02	96	89	75
Pow5	27	22	6,5	0,24	82	42	18
Pow6	35	23,4	8,4	0,24	83	49	26

Table S3A - WGS data without methylation digest with the McrBc enzyme.

Sample	With methylation digest						
	Reads (m)	% of mapped reads	Mean coverage	Normalized coverage	Percentage of genome with		
					1X coverage	5X coverage	10X coverage
Poc1	10	89,6	35	3,50	94	82	67
Poc2	29	55,3	47	1,62	96	92	85
Poc3	25	77,4	65,7	2,63	96	91	83
Poc4	19	82,1	48,5	2,55	94	83	67
Poc5	49	77,2	115	2,35	95	79	61
Pow1	26,4	95,2	91	3,45	94	87	82
Pow2	23	92,7	75,2	3,27	95	91	85
Pow3	27	97,7	92,6	3,43	95	92	88
Pow4	31	67,5	67,5	2,18	95	92	90

Pow5	31	95	102,6	3,31	95	92	91
Pow6	38	58,9	67,7	1,78	96	92	89

Table S3B - WGS data with methylation digest with the McrBC enzyme.

Species	Gene name	Gene ID	Chr.	Position	Effect	Ref	Alt	Change in codon	Change in amino acid
P. ovale curtisi	*Poccrt*	PocGH01_01016900	1	324,055	Intronic	T	A	/	/
	Pocdhfr	PocGH01_05028400	5	762,499	Non-synonymous	G	T	Gcg/Tcg	**A15S**
				762,628	Non-synonymous	A	C	Agt/Cgt	**S58R**
				762,749	Non-synonymous	A	C	cAt/cCt	**H98P**
				763,488	Synonymous	T	C	taT/taC	T344T
	Pocmdr1	PocGH01_10018700	10	323,426	Non-synonymous	A	G	Att/Gtt	**I1303V**
	Pocdhps	PocGH01_14036800	14	1,121,715	Non-synonymous	A	G	Aaa/Gaa	**K189E**
				1,121,456	Non-synonymous	A	G	gAc/gGc	**D275G**
P. ovale wallikeri	*Powcrt*	PowCR01_010011800	1	331,693	Non-synonymous	T	G	Tgt/Ggt	**C19G**
				333,150	Non-synonymous	G	T	ttG/ttT	**L216F**
	Powdhfr	PowCR01_050023500	5	843,985	Non-synonymous	T	G	ttT/ttG	**F57L**
				843,986	Non-synonymous	A	C	Agt/Cgt	**S58R**
				844,152	Non-synonymous	G	A	aGc/aAc	**S113N**
	Powmdr1	PowCR01_100013900	10	359,931	Non-synonymous	T	A	Ttc/Aac	**F34N**
	Powdhps	PowCR01_140031200	14	1,123,447	Synonymous	A	G	aaA/aaG	L195L
				1,123,710	Synonymous	T	C	taT/taC	T106T

Table S6. SNPs detected in *P. ovale curtisi* or *P. ovale wallikeri* genes orthologous to major *P. falciparum* drug resistance genes.
